# Antimicrobial Natural Hydrogels in Biomedicine: Properties, Applications, and Challenges—A Concise Review

**DOI:** 10.3390/ijms24032191

**Published:** 2023-01-22

**Authors:** Oliwia Kapusta, Anna Jarosz, Katarzyna Stadnik, Dimitrios A. Giannakoudakis, Bartłomiej Barczyński, Mariusz Barczak

**Affiliations:** 1 Institute of Chemical Sciences, Faculty of Chemistry, Maria Curie-Sklodowska University, 20031 Lublin, Poland; 2 Department of Chemistry, Aristotle University of Thessaloniki, 54124 Thessaloniki, Greece; 3 1st Department of Oncological Gynecology and Gynecology, Medical University in Lublin, 20-059 Lublin, Poland

**Keywords:** hydrogels, antibacterial properties, wound healing, tissue regeneration, biomedical applications

## Abstract

Natural hydrogels are widely used as biomedical materials in many areas, including drug delivery, tissue scaffolds, and particularly wound dressings, where they can act as an antimicrobial factor lowering the risk of microbial infections, which are serious health problems, especially with respect to wound healing. In this review article, a number of promising strategies in the development of hydrogels with biocidal properties, particularly those originating from natural polymers, are briefly summarized and concisely discussed. Common strategies to design and fabricate hydrogels with intrinsic or stimuli-triggered antibacterial activity are exemplified, and the mechanisms lying behind these properties are also discussed. Finally, practical antibacterial applications are also considered while discussing the current challenges and perspectives.

## 1. Introduction

Hydrogels are three-dimensional, hydrophilic networks formed by flexible polymer chains swollen by water or biological fluids and able to store a large amount of it while maintaining the 3D structure that can be cast into practically any shape or form [1]. Hydrogels can be divided into two groups: natural hydrogels (e.g., alginate, collagen, fibrin, hyaluronic acid, chitosan, agarose or starch) and synthetic hydrogels (e.g., poly (ethylene glycol), poly(vinyl alcohol) or polymethyl methacrylate). Although having some drawbacks related to their not sufficient processing reproducibly and changeable composition, natural hydrogels exhibit high bioaffinity and biocompatibility.

**Figure 1:**
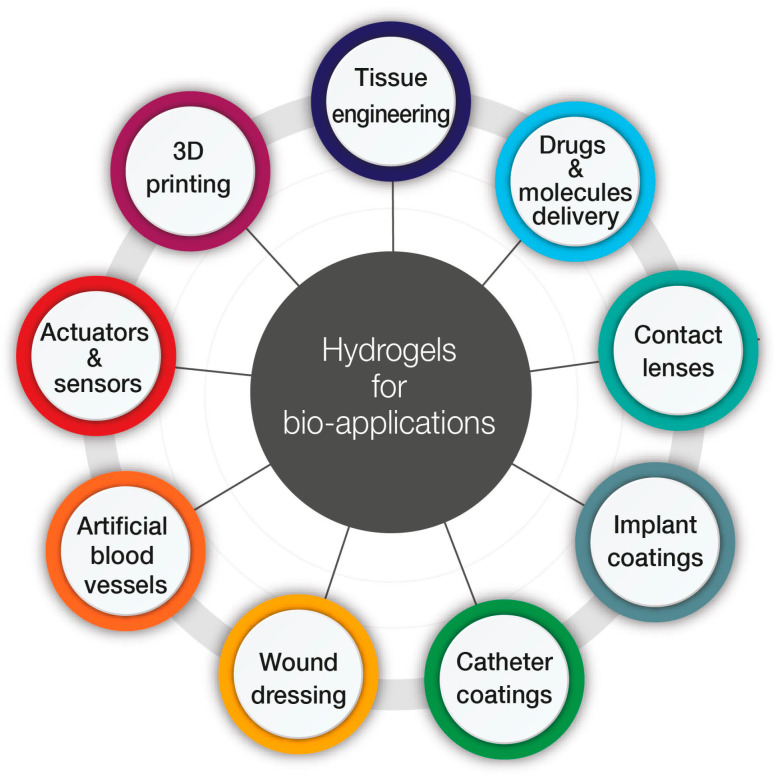
Application of hydrogels in biomedicine.

Biomedical applications of hydrogels have constantly been evolving during the last years, driven by discoveries in the field of biology and chemistry. Compared with other types of biomaterials, hydrogels have increased biocompatibility, tunable biodegradability, and tunable porous structure and thus permeability; their disadvantages are mainly associated with low mechanical strength and fragile nature [2]. Novel strategies to construct these materials led to achieving more and more complex and functional hydrogels, including composite hydrogels containing a plethora of various nano/microstructures. Therefore, these advantageous materials are finding increasing application in many biomedical fields, including drug delivery [3,4,5,6], tissue engineering [7,8,9], 3D printing [10,11,12,13,14], and, more recently, in biosensing and actuating applications [15,16,17]. The most promising applications of hydrogels are summarized in Figure 1.

Still, one of the most prominent applications of hydrogels is related to wound healing [18,19,20,21,22], where they serve as advanced “moisture donor” dressings, increasing collagenase production and facilitating autolytic debridement [23]. Anyway, the use of hydrogels facilitates oxygen transmission to the wounds and allows for absorbing and retention of exudate within the gel mass [24]. In addition to these extremely useful advantages in the treatment of wounds, hydrogels can have one more crucial from the point of view of this literature review: they can provide antimicrobial action preventing or slowing down the development of microbial infections, which are one of the major problem related to wound healing constituting one of the most prominent factors preventing wounds from healing, especially chronic ones, requiring challenging treatment [25]. Natural hydrogels are considered extremely useful biomaterials due to their effective inhibition of bacterial infections.

Bacterial wound infection develops through three consecutive phases [26]:

(i) contamination—bacteria are transferred into the wound from the surrounding environment,
(ii) colonization—bacteria are replicated and start to adhere to the wound-forming biofilm, however without interfering with the ongoing wound healing process,
(iii) infection—the replication rate of bacteria begins to overload the immune system leading to various consequences.

Some common Gram-negative, as well as Gram-positive bacterial pathogens that have been isolated from infected wounds, include *Staphylococcus aureus, Pseudomonas aeruginosa*, *Proteus mirabilis*, *Escherichia coli*, *Corynebacterium spp.*, *Acinetobacter baumannii*, *Anaerobic Cocci* [27,28]. It is worth noting that wound infections are often found to be polymicrobial.

The porous structure of hydrogels—possible to be controlled by tuning the crosslinking density—can provide a matrix for loading of antimicrobial factors (antibiotics, extracts, nanoparticles, etc.) and, at the same time allow for tuning the release rate (depending on the diffusion coefficient of the factor through the gel network [2,29]) of the antimicrobial agent. Concurrently, biocompatibility and biodegradability can also be designed to prove the high potential of natural hydrogels to be used as antimicrobial biomaterials, particularly wound dressings.

This review is an up-to-date compilation of the latest information and reports that appeared in the literature on the biocidal properties of various types of natural hydrogels. What makes it a handful and useful is a collection of over 250 carefully selected articles on the biocidal properties of hydrogels, in the way that the review can serve as a source of knowledge for both specialized scientists and students starting their exploration of these issues.

## 2. Design, Synthesis, and Properties of Antimicrobial Hydrogels

### 2.1. Hydrogels with Intrinsic Antimicrobial Activity

The role of each of the discussed hydrogels in the wound healing process is a complex function of many interactions, including stimulation of cell proliferation and angiogenesis, activation of macrophages and neutrophils to initiate the healing process, inhibition of metalloproteinases, regulation of the oxidation-reduction environment or increase in microbiological purity. Unfortunately, to this day, the exact description of the bactericidal properties of the hydrogels themselves is not fully explained. Since hydrogels can either be intrinsically antibacterial or be doped with antibacterial components, it is worth taking a moment to look at the intrinsic antimicrobial properties of non-doped hydrogels.

#### 2.1.1. Polysaccharide Hydrogels

**Figure 2:**
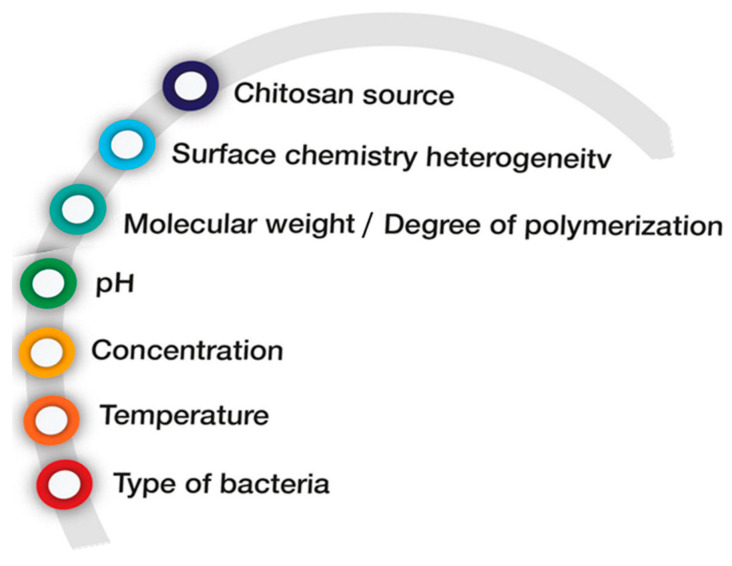
Intrinsic and external factors influencing the antibacterial activity of chitosan.

Among the group of polysaccharide hydrogels, there is one prominent and most frequently studied example—chitosan, in which proper bactericidal effect is widely used. Chitosan is a natural polymer obtained from chitin (which is the building block of crustaceans and insects) through enzymatic or chemical deacetylation. Both chitin and chitosan, owning to their excellent biochemical properties such as biocompatibility, biodegradability, non-toxicity, and processability, have found many promising biomedical applications [30]. Due to the presence of amine groups, chitosan polymers are positively charged at pH below 6 and thus can interact electrostatically with negatively charged regions on the microbial membrane [31]. In this way, chitosan can bind and disrupt the normal functions of the bacterial membrane by provoking the leakage of intracellular components as well as inhibiting the transport of nutrients into the cells [32,33,34,35]. Some studies reported the dependency of selected antibacterial and antifungal activities from both intrinsic physicochemical properties of chitosan (e.g., molecular weight, degree of deacetylation, hydrophobicity) and external factors (pH of medium, concentration, type of bacteria) [36,37,38]. These factors are shown in Figure 2.

However, it should be noted that apart from the above-mentioned mode of interaction (involving the electrostatic attraction of cationic groups of chitosan with negatively charged zones on the bacterial surface), other possible mechanisms of antibacterial action have also been proposed [31]. In the case of Gram-negative bacteria, it was suggested that chitosan could affect the permeability of the outer envelope by forming ionic bonds, which prevent the transport of nutrients into the cell and increase internal osmotic pressure [39]. Another proposed mechanism assumes that chitosan is able to penetrate inside the microbial cell and interact with DNA intracellularly, halting its transcription and RNA synthesis. In this scenario, chitosan can penetrate various types of membranes, including murein cross-linked walls as well as cytoplasmic membranes, and thus its cell-destroying efficiency would depend on the molecular weight [40]. This mechanism, although it seems interesting, should now be considered speculative as it has not been unequivocally confirmed. Another mechanism is based on the chelating capabilities of chitosan, which explains its activity at pH above 6, where free (non-protonated) amino groups can form bonds with metals such as Ca^2+^ or Mg^2+^ present in the cell walls. Formed bonds block the production of toxins and thus inhibit the overall growth of microorganisms. In conclusion, it should be clearly noted that among the above-mentioned mechanisms of chitosan action, the most frequently cited and suggested mechanism is the interaction of chitosan with the outer surface of bacteria or fungi as a result of electrostatic attraction, followed by cracking of the surface and the release of intracellular components [41,42].

An injectable hydrogel based on two natural polymers, chitosan and konjac glucomannan, linked together via Schiff base linkages showed good antibacterial activity against Gram-positive *Staphylococcus aureus* and Gram-negative *Escherichia coli* bacteria with 96% and 98% killing efficiency, respectively [43]. Chitosan/bacterial cellulose semi-interpenetrating hydrogels were successfully prepared by mixing both components and their subsequent cross-linking with glutaraldehyde. The resulting hydrogels showed antibacterial properties against the same bacteria as before, and the antibacterial properties were dependent on the chitosan-to-cellulose ratio [44].

Due to its bactericidal properties, low toxicity, and high biocompatibility, chitosan was approved as a food additive many decades ago—its application in this area has been thoroughly reviewed by M. Kong et al. [41]. Biomedical uses of chitosan (and particularly chitosan-based hydrogels) as antibacterial materials have also emerged over time and were mostly related to wound dressing, tissue engineering, and drug delivery. However, in the most currently tested applications, chitosan hydrogels are always doped with various types of agents, which will be discussed in the next parts of the article. The main disadvantages of chitosan are its insolubility in a neutral (so often close to physiological) aqueous medium and insufficient antibacterial activity, which hinders its use as an effective antimicrobial agent [45]. Nevertheless, the intention of the authors was to note the fact that chitosan itself is also a bactericidal material, regardless of whether its effect is enhanced (sometimes tremendously) by the addition of various types of active substances.

One of the strategies is to introduce quaternary ammonium groups into various natural hydrogels as these groups have intrinsic bacterial membrane disruption activity, therefore, can be used for enhancing the antibacterial performance. There is an interesting review published recently describing this approach in a comprehensive manner [45].

In addition to chitosan, there are other polysaccharide-based hydrogels with antibacterial properties, such as for example gelatin and hyaluronic acid. Gelatin, a product derived from the hydrolysis of collagen, is widely used in biomedical applications (e.g., drug release, wound dressings, cell culture, and scaffolds for tissue engineering) because of its good biocompatibility, biodegradability, and non-immunogenicity [46]. In addition, gelatin contains arginine-glycine-asparagine (RGD) units, which are known to promote cell adhesion, migration, and proliferation [47]. Hyaluronic acid is a natural linear polysaccharide found in numerous tissues (including the extracellular matrix), playing an important role in cellular signaling and wound healing [48] as it prevents the proliferation of bacteria and acts as an anti-inflammatory agent.

#### 2.1.2. Antimicrobial Peptide Hydrogels

Antimicrobial peptides are a group of small natural (produced by multicellular organisms) or synthetic polypeptide molecules. They play the role of the innate immune system of various organisms [49,50,51]. Therefore, they are an excellent platform for creating natural hydrogels with antibacterial properties. Currently, at least several hundred proteins of this type have been discovered [52] being excellent drug candidates for clinical exploitation due to their numerous advantages, including biocompatibility, biodegradability, and ease of synthesis and modification. The antibacterial mechanism of antimicrobial peptides is different from that of antibiotics. Therefore it is much more difficult for bacteria to produce drug resistance [53].

Some antimicrobial peptides may self-assemble by themselves into supramolecular hydrogels, which usually enhance their antimicrobial ability [50]. For example, the high inherent antibacterial activity of a β-hairpin based peptide hydrogel has been demonstrated by Sallick et al. [51]. During the experiments, gels were proved to be effective against a wide range of pathogens, including Gram-positive (*Staphylococcus epidermidis, Staphylococcus aureus,* and *Streptococcus pyogenes*) and Gram-negative (*Klebsiella pneumoniae* and *Escherichia coli*) bacteria. The suggested mechanism of antibacterial action based on membrane disruption leading to cell death upon cellular contact with the gel surface was proposed [51].

Usually, antimicrobial peptides are covalently connected to various hydrogelator fragments/molecules and—after gelation –form an integral part of the resulting hydrogel. Fluorenylmethyloxycarbonyl (Fmoc) group is widely used as a capping group for the generation of short peptide-based hydrogelators; some of them are already commercially available. Long-range aromatic stabilization via π–π stacking of Fmoc groups is a major driving force promoting the gelation of these peptides [54]. For example, Schnaider et al. obtained a diphenylalanine peptide that, upon self-assembling, showed high antibacterial activity against *Escherichia coli*. It was shown that nano-assemblies completely inhibited bacterial growth, triggered upregulation of stress-response regulons, induced substantial disruption to bacterial morphology, and caused membrane permeation and depolarization [55]. Later, McCloskey et al. designed various ultrashort Fmoc-peptide hydrogelators/peptides able to form soft hydrogels. The majority of the fabricated supramolecular hydrogels demonstrated selective action against biofilms of Gram-positive (*Staphylococcus aureus*, *Staphylococcus epidermidis*) and Gram-negative (*Escherichia coli*, *Pseudomonas aeruginosa*) pathogens, proving a high potential for their use in biomaterial applications, including antibacterial agents [56]. Porter at al. showed that modification of terminal functional groups to the amino or carboxylic acid or both could affect the antibacterial selectivity against *Staphylococcus aureus* [57]. Interestingly, functionalization of Fmoc-protected dipeptides with pyridinium nitrogen at the C-terminal may provide a class of positively charged hydrogelators forming supramolecular hydrogels at low concentrations (∼0.6%) and show remarkable bactericidal efficacy against both Gram-positive (*Bacillus subtilis* and *Staphylococcus aureus*) and Gram-negative bacteria (*Pseudomonas aeruginosa* and *Escherichia coli*) [58]. Finally, it should also be taken into account that in some cases, antimicrobial resistance has already been observed, including intrinsic resistance to antimicrobial peptides by the exposure of positively charged lipids on their membrane [59].

### 2.2. Hydrogels Loaded with Antimicrobial Agents

When loaded with antimicrobial agents, hydrogels can tremendously increase their antimicrobial efficacy. Among antimicrobial agents/factors which can be loaded into a hydrogel matrix, four main groups can be considered: antibiotics, biological extracts, nanoparticles, and antimicrobial peptides, which are separately discussed in this chapter.

#### 2.2.1. Hydrogels Loaded with Antibiotics

The biocompatible nature of most natural hydrogels makes them a convenient starting point to engineer a wide range of antimicrobial systems based on various active molecules, among which the most commonly used group are, of course, antibiotics [60]. Antibiotics can fight bacterial infections by killing bacteria (bactericidal effect) or slowing or stopping their growth (bacteriostatic effect) [61,62], acting selectively in various concentrations and affecting the cellular structures and the metabolic processes of various microorganisms [61,63]. The controlled hydrophilic/hydrophobic nature of many natural hydrogels provides a good environment for the incorporation of various small molecules, including antibiotics. Particularly, local antibiotic therapy is increasingly being recognized for its role in preventing and treating bacterial infections. Medical dressing for topical use (mainly wound treatment) based on antibiotic-loaded hydrogels has become more and more popular and relevant as they can provide multiple properties simultaneously [64,65]. Bacterial infections impede the wound-healing process, leading to complications such as chronic wounds and ulcerations [66].

Antibiotics that are most commonly used for wound healing can be divided into several categories [67]:

1. Aminoglycosides (e.g., gentamicin, streptomycin) and tetracyclines (e.g., doxycycline, tetracycline hydrochloride) block the pathway of protein synthesis,
2. Beta-lactams (e.g., ampicillin, ceftadizime, cefazolin) and glycopeptides (e.g., vancomycin) inhibit the synthesis of the bacterial cell wall,
3. Sulphonamides (e.g., sulfadiazine) which interfere with the synthesis of the key metabolites,
4. Quinolones (e.g., ciprofloxacin, levofloxacin) inhibit the synthesis of nucleic acids.

**Figure 3:**
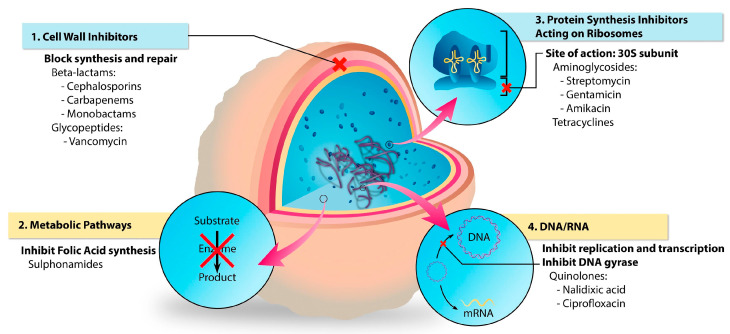
Representation of the different targets of antibiotics within bacteria. Reprinted with permission from [68]. Copyright 2022, Elsevier.

The incorporated antibiotics can act via four main mechanisms, either affecting bacteria structure or their metabolic pathways; these mechanisms are presented in Figure 3 [68].

Regardless of the type of antibiotic, the healing efficiency of an antibacterial wound dressing that delivers antibiotics depends on the drug release profile, the physicochemical properties of the drug, and the type and properties of the hydrogel matrix. However, for an antibiotic to be effective, it must reach the concentration necessary to inhibit the growth or kill the pathogen at the site of infection while remaining safe for human cells [68]. The hydrophilic nature of the hydrogel provides such a suitable substrate for the storage and controlled release of small molecules of antibiotic formulation, such as gentamicin [58], ciprofloxacin [59], vancomycin [60], amoxicillin [61] as the most popular representatives of antibiotics loaded into various hydrogel matrixes, and are discussed below separately.

##### Gentamicin

Gentamicin is one of many aminoglycoside antibiotics commonly recommended by doctors and medical specialists worldwide [69,70]. It is a traditional broad-spectrum aminoglycoside antibiotic used to treat skin, soft tissue, and wound infections [71,72,73]. The antibiotic shows the greatest effectiveness against aerobic Gram-negative but is also used in conjunction with other antibiotics to treat infections caused by Gram-positive and Gram-negative organisms (e.g., *Staphylococcus aureus, Streptococci, Escherichia coli, Pseudomonas aeruginosa*) [74,75]. This antibiotic penetrates inside the bacterial cell and there—by binding with the bacterial ribosome—creates abnormal bacterial proteins, the incorporation of which into the cell membrane results in an increase in its permeability and cell death [76,77].

Biodegradable hybrid wound dressings loaded with gentamicin have been studied to evaluate gentamicin release and in vivo wound healing using a guinea pig burn model, compared to the neutral non-adherent dressing material Melolin^®^ and Aquacel^®^ Ag. It was concluded that the obtained hydrogel-based dressings exhibited promising results and did not require frequent bandage changes [78].

Hydrogel wound dressing consisting of hyaluronic acid cross-linked with gentamicin via EDC/NHS bioconjugation strategy. This hydrogel was able to treat bacterial infection locally. Tuning crosslinking density was important to control a sustained release of gentamicin for up to nine days with a range of adhesive and cohesive properties [48].

The antibacterial efficiency of hydrogels based on pullulan (extracellular microbial polysaccharide produced by different strains of *Aureobasidium*) and cysteamine with incorporated gentamycin have been evaluated by Li et al. [79]. Based on the release of gentamycin and the inhibition of bacterial proliferation against *Staphylococcus aureus* and *Escherichia coli*, it was hypothesized that antibiotics containing hydrogels could protect the wound surface from bacterial invasion.

Bakhsheshi-Rad et al. prepared electrospinning-derived chitosan-alginate nanofibers as a platform for releasing gentamicin. The nanofibers loaded with gentamicin displayed superior antibacterial activity compared with the nanofibers having a lower amount of gentamicin. The results proved that gentamicin-loaded chitosan-alginate nanofibers have the potential to be used in the future as antibacterial wound dressing, including their ability of gentamicin delivery through the wound area [80].

##### Ciprofloxacin

Ciprofloxacin is a chemotherapeutic agent from the fluoroquinolone group [81,82]. It works against Gram-positive and Gram-negative bacteria [83,84,85]. Its mechanism of action is based on the inhibition of two bacterial enzymes which are involved in the replication, transcription, and recombination of bacterial DNA: topoisomerase type II (DNA gyrase) and topoisomerase IV [86,87]. Since disruption of these processes leads to bacterial death, ciprofloxacin is a bactericidal drug [88].

Hydrogels fabricated by cross-linking of chitosan with bifunctional PEG glyoxylic aldehyde were loaded with ciprofloxacin and provided its sustained release for up to 24 h (≥80%) and displayed efficient activities (≥80%) against *Escherichia coli* for up to 12 h. The hydrogels were demonstrated to be nontoxic and cytocompatible toward mammalian cells (NIH-3T3) [66].

Biocompatible and degradable dual drug-delivery systems based on hyperbranched copolymers obtained via thiol-ene click chemistry were proposed. It was demonstrated that the simultaneous delivery of two antibiotics might be achieved, despite the fact that hydrophilic novobiocin is entrapped within the hydrophilic hydrogel, while the hydrophobic ciprofloxacin is encapsulated within the dendritic nanogels. Such hybrid hydrogels enable the quick release of novobiocin and prolonged release of ciprofloxacin. In vitro cell infection assays demonstrated that the antibiotic-loaded hybrid hydrogels can be used to treat bacterial infections, exhibiting better antibacterial activity against *Staphylococcus aureus* when compared with commercial antimicrobial band aids [89].

Cacicedo et al. prepared bacterial cellulose-chitosan films for ciprofloxacin delivery. The incorporation of ciprofloxacin into hybrid cellulose-chitosan films enabled its sustained release for more than 6 h. The presence of chitosan allowed for a slower controlled release of the antibiotic when compared with plain bacterial cellulose. The ciprofloxacin-loaded films demonstrated a good antibacterial performance against *Pseudomonas aeruginosa* and *Staphylococcus aureus*. In this case, a synergic effect of chitosan and ciprofloxacin antimicrobial activity was observed; in addition in vitro studies revealed the lack of cytotoxicity of the obtained hydrogel films in human fibroblasts [90].

The development of an antibacterial bioadhesive hydrogel with the addition of micelles containing ciprofloxacin has become a solution to the problem. Analysis of the release profile provided information on the effectiveness of the action in the first 24 h of use. Hydrogels doped with ciprofloxacin showed excellent antibacterial properties against *Pseudomonas aeruginosa* and *Staphylococcus aureus* [64]. The release of ciprofloxacin from trimethyl chitosan (TMC)/sodium carboxymethyl xanthan gum (CMXG) hydrogel slowing multiplication of Gram-positive and Gram-negative bacterial strains characterized by a larger degree of inhibition zone as compared to ampicillin or gentamicin [91].

Antibacterial bioadhesive hydrogel loaded with micelles containing ciprofloxacin for the management of corneal injuries was reported by Khalil et al. [92]. The corneal cells treated with the hydrogel were characterized by a decrease in colony-forming units (CFU) and a higher corneal epithelial viability after 24 h as compared to non-treated corneas and corneas treated with hydrogel without ciprofloxacin. It was concluded that the fabricated hydrogel presents a promising suture-free solution to seal corneal wounds while preventing infection.

It was also demonstrated that ciprofloxacin could be self-assembled with a hydrophobic tripeptide (Leu-Phe-Phe) into antibacterial nanostructured hydrogels with high drug loading efficiency (2 mg ml^−1^ and 30% w/w) and a prolonged release while its antimicrobial activity against *Staphylococcus aureus*, *Escherichia coli*, and *Klebsiella pneumoniae* can be achieved [93].

##### Vancomycin

Vancomycin is a glycopeptide antibiotic with a bactericidal action [94] mainly used in the case of serious *Staphylococcal* and *Streptococcal* infections in patients who are resistant or allergic to penicillins and cephalosporins [95]. The mechanism of action is based on blocking cell wall biosynthesis by inhibiting peptidoglycan polymerization by binding directly to D-alanyl-D-alanine terminal peptides and inhibiting cross-linking by transpeptidase [96]. Additionally, it influences the permeability of cell membranes and RNA synthesis. This antibiotic is mainly aimed at inhibiting the multiplication of Gram-positive bacteria, while strains of Gram-negative bacteria are resistant to this drug and are not able to interfere with the barrier—the cell wall [96].

Hyaluronic acid hydrogel loaded with vancomycin and gentamicin was synthesized and used for the eradication of chronic methicillin-resistant *Staphylococcus aureus* orthopedic infections using a mammal model (sheep). The increased efficacy was attributed to two important features, drug release pharmacokinetics and hydrogel’s degradability [97].

Similarly, hyaluronic acid hydrogels loaded with various antibacterial agents (cefuroxime, tetracycline, amoxicillin, and acetylsalicylic acid) were tested as antibacterial and anti-inflammatory agents against *Staphylococcus aureus*. Drug-loaded hydrogels were demonstrated to have remarkable antibacterial and anti-inflammatory activities by their significant (*p* < 0.05) reduction in both *Staphylococcus aureus* bacterial infection and the levels of painful TNF-α, IL-6, and IL-8 pro-inflammatory cytokines [98].

Posadowska et al. fabricated an injectable gellan gum-based nanoparticles-loaded system for the local delivery of vancomycin in osteomyelitis treatment [99]. The obtained material was shown to be a biocompatible system displaying easiness of injection at low extrusion force, self-healing ability after disruption, adjustable vancomycin release, and antimicrobial properties against Gram-positive bacteria *Staphylococcus aureus* and *Staphylococcus epidermidis*.

##### Amoxicillin

Amoxicillin is a beta-lactam antibiotic derived from penicillin. It is active against Gram-positive bacteria, including nonpenicillin-resistant streptococcal, staphylococcal, and enterococcal species. It also has activity against some Gram-negative bacteria, including *Helicobacter pylori*, as well as anaerobic organisms [100]. Chang et al. reported chitosan/poly-γ-glutamic acid nanoparticles incorporated into pH-sensitive hydrogels as an efficient carrier for amoxicillin delivery. It was shown that nanoparticles could penetrate cell−cell junctions and interact with *Helicobacter pylori*-infected sites in the intercellular spaces. Additionally, the composite hydrogel protected amoxicillin from the actions of gastric juice [101].

In a more recent study, starch-based composite hydrogel loaded with amoxicillin was tested as a potential platform for the controlled release of amoxicillin and inhibition of bacterial growth. In vitro bacterial growth inhibition was verified via disc diffusion assay against bacteria of clinical interest, such as *Escherichia coli*, *Staphylococcus aureus,* and *Pseudomonas aeruginosa*. Significant bacteria growth inhibition effects of amoxicillin-loaded hydrogels were evidenced, revealing the future potential for oral administration and the local treatment of bacterial infections [102].

Chitosan-alginate hydrogels prepared by a simple, solvent-free approach for simultaneous and sustained releases of three antibiotics (ciprofloxacin, amoxicillin, and vancomycin) have been most recently reported by Khan et al. The antibiotics were loaded during the gelation process to maximize loading efficiency. The results revealed that the physicochemical properties of the combination of the drugs-loaded play an essential role not only in the morphology, structural changes, and release profiles but also in antibacterial activity [103].

Chitosan-PEG hydrogels exhibiting biocompatible, antibacterial, anti-inflammatory, and self-healing properties were obtained by del Olmo et al. Antibacterial activity was evaluated against *Staphylococcus aureus* and *Escherichia coli*, which are common microorganisms identified in infected wounds. Hydrogels were loaded with various antibiotics, including cefuroxime, tetracycline amoxicillin antibiotics, as well as acetylsalicylic acid for the subsequent sustainable release. All the drugs were confirmed to enhance the antibacterial and anti-inflammatory activity [104]. The same research group demonstrated similar outcomes using an even simpler hydrogel formulation, i.e., based on chitosan crosslinked with genipin [105].

##### Other Antibiotics

Various cefotaxime sodium-loaded hydrogel formulations (including pectin, xanthan gum, and guar gum) were prepared, characterized, and tested against wound pathogens such as *Staphylococcus aureus*, *Escherichia coli*, and *Pseudomonas aeruginosa*, using either pure drug or Fucidin^®^ cream as control. Further, in vivo studies were performed to demonstrate the efficacy of eradication of Gram-negative (*Pseudomonas aeruginosa*) or Gram-positive (methicillin-resistant *Staphylococcus aureus*) bacteria from an infected rat wound model. It was shown that some cefotaxime gel formulations could be a very promising and innovative topical alternative for the treatment of skin infections caused by Cefotaxime-susceptible bacteria [106].

Gelatin methacryloyl hydrogels have been fabricated and tested for the localized delivery of cefazolin. It was demonstrated that cefazolin delivered from the hydrogel induced a dose-dependent antibacterial efficacy against *Staphylococcus aureus*, chosen as a model bacteria [107].

A photo-cross-linked hydrogel based on gelatin served as a platform designed for ceftriaxone release. Antibacterial testing carried out on the hydrogel system by the disc diffusion method resulted in the inhibition of *Staphylococcus aureus* strains [108].

Alginate hydrogels (in the form of loaded alginate microparticles or films) incorporating neomycin or propolis were tested as potential dressings for diabetic ulcers. Microbial penetration tests revealed that all the hydrogels were able to act as barriers to the penetration of microorganisms, which is an important characteristic of wound dressings [109]. In another study, neomycin was incorporated into composite hydrogels composed of poly (vinyl alcohol) (PVA), carboxymethyl cellulose, and cellulose nanofibers. The resulting hydrogel exhibited improved biodegradability, biocompatibility, pH-responsiveness, and effectivity against *Escherichia coli* and *Staphylococcus aureus*, which was attributed to the controllable release of neomycin [110].

The sustained release of levofloxacin from thermosensitive chitosan-based hydrogel was reported by Cheng et al. [111]. Antibacterial activity of levofloxacin-containing hydrogel against *Staphylococcus aureus* and *Staphylococcus epidermidis*, chosen as two common aerobic bacteria found in wound infections of postoperative endophthalmitis. The results of antibacterial studies demonstrated a long-term antibacterial property of the developed levofloxacin-doped hydrogel. The same group has developed another chitosan -based hydrogel loaded with levofloxacin for the topical treatment of keratitis (i.e., inflammation of the eye’s cornea). This time, *Staphylococcus aureus* was selected as a model bacterial threat to check whether the obtained hydrogels could effectively fight them. It was demonstrated that the obtained hydrogel significantly inhibited bacterial growth indeed, with no histological evidence of the bacteria in the studied ex vivo rabbit model of keratitis. Moreover, the hydrogel exhibited remarkable anti-inflammatory effects and sustained release of levofloxacin [112]. In another study, the delivery of levofloxacin from hyaluronic acid nanohydrogels for the treatment of bacterial intracellular infections studied by Montanari et al. The minimal inhibitory concentration values for *Staphylococcus aureus* and *Pseudomonas aeruginosa* were compared with pure and hydrogel-loaded levofloxacin-loaded nanohydrogels indicating the increase in antibacterial efficacy of the latter. An additional advantage from the point of view of in vivo applications is the possibility of ease sterilization of the obtained materials without losing the desired properties [113].

Multicomponent chitosan-based fabricated by free radical graft copolymerization and loaded with clarithromycin. In vitro drug release assessments demonstrated that clarithromycin can be sustainably released in a simulated gastric medium (pH = 1.2) for a prolonged period of time. It was concluded that the obtained multi-composite could be used as an effective drug delivery system to cure *Helicobacter pylori*-related infection. However, no antibacterial tests were performed [114].

A Doxycycline drug delivery platform based on carboxymethyl chitosan conjugated with caffeic acid and its composite with polyacrylamide was reported by Moghaddam et al. The cytotoxicity results indicated that the synthesized hydrogels are relatively nontoxic, and the bacterial inhibition zones around doxycycline-loaded hydrogels against *Staphylococcus aureus* and *Escherichiacoli* demonstrated their antibacterial action [115].

Composite gelatin-based injectable antimicrobial conductive hydrogels for wound disinfection and infectious wound healing were thoroughly studied by Liang et al. against methicillin-resistant *Staphylococcus aureus*. The very detailed biomedical evaluation was run using a plethora of approaches by use of an infected mouse model: the wound closure rate, the length of dermal tissue gap, number of blood vessels and hair follicles in hematoxylin-eosin staining, the related cytokines and regeneration of blood vessels in immunofluorescence were all further studied. All the results demonstrated the superior wound healing effect of the synthesized doxycycline-loaded hydrogel (but also its drug-free analog) in infectious skin tissue defect repair, indicating their great potential for infected wound healing [116].

Wound dressing application of chitosan-gelatin hydrogels embedded with ampicillin-loaded hyaluronic acid nanoparticles was investigated by Özkahraman et al. In vitro ampicillin release studies showed that more than half of the antibiotic load was released from the hydrogels after five days, but most of that amount after five hours. The antibacterial performance of hydrogels against *Staphylococcus aureus* and *Escherichia coli* was demonstrated using an agar disc diffusion test [117].

**Figure 4:**
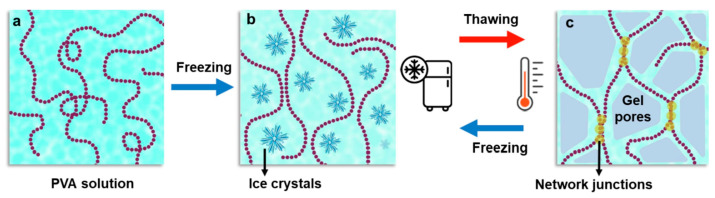
Preparation of hydrogels by the freezing-thawing method. (**a**) polymeric chains in solution, (**b**) freezing step leading to entrapment of polymeric chains between ice crystals due to phase separation, (**c**) the gel network is formed as the ice crystals are forming hydrogel pores. Reprinted with permission from [119]. Copyright 2022, Elsevier.

Composite hydrogel composed of poly(vinyl alcohol), egg white (matrix), and montmorillonite nanoclay (reinforcement) was fabricated by Delir et al. by freeze-thaw technique. In vitro, clindamycin release studies showed the cumulative fractional release of clindamycin was decreased either by increasing the weight percentage of the incorporated montmorillonite into the wound dressings or by decreasing the pH of the release medium. It was confirmed that more than 90% of *Staphylococcus aureus* bacteria could be eliminated using the clindamycin-loaded hydrogel [118]. It is worth mentioning here that freezing-thawing is one of the frequently used approaches (also commercial) to fabricate particularly PVA-based hydrogels as PVA gelation through repeated freezing-thawing cycles occurs without an externally added crosslinking agent, resulting in ultrapure hydrogels [119]. The schematic idea of this method is shown in Figure 4.

Another PVA-based hydrogel prepared by the freeze-thaw technique was obtained by Qing et al. [120]. Apart from PVA, it was composed of N-succinyl chitosan and loaded with lincomycin (which, similarly to clindamycin, belongs to the lincosamides [121]). The incorporation of this antibiotic resulted in a remarkable antibacterial activity against *Escherichia coli* and *Staphylococcus aureus* (78% of the latter was inhibited with 75 μg/mL lincomycin), with more than 74 % of the drug released within 24 h. The nontoxic nature of the composite hydrogels was also shown using an MTT assay suggesting the promising potential of the obtained hydrogels for wound dressing [120].

Gelatin-polyacrylamide hydrogel loaded with tetracycline hydrochloride was demonstrated to have high antibacterial activity against *Staphylococcus aureus* and *Escherichia coli,* reducing the wound area (96%) better than the antibiotic-free hydrogel component (86%) after 14 days [122].

Silk fibroin/sodium alginate hydrogel scaffolds loaded with teicoplanin (semisynthetic glycopeptide antibiotic with similar activity to vancomycin) and additionally, phenamil (a blocker of Na^+^ translocation) were evaluated in a rat model of chronic bone infection. The released antibiotic maintained its high antibacterial activity (>75%) against methicillin-resistant *Staphylococcus aureus* for 35 days and in various physiologically relevant pH environments (5.5, 7.4, and 8.5). The obtained results indicated that the fabricated scaffolds could eradicate the infection and, at the same time, improve bone regeneration [123].

Chitosan-based hydrogel loaded with whey protein-based microsized particles containing chloramphenicol have been demonstrated to have remarkable ex vivo antibacterial activity against *Staphylococcus aureus* with doubling antibacterial activity after 24 h, comparing chloramphenicol-loaded and chloramphenicol-free samples [124].

Alginate/hyaluronic acid-based hydrogels were modified with grafting phenylboronic acid to the alginate side chain and have shown antibacterial and anti-inflammatory properties as a result of the effective loading of amikacin (aminoglycoside antibiotic often used for treating severe infections caused by various Gram-negative multidrug-resistant bacteria) and naproxen. Both drugs were pre-loaded into the micelles, preserving the structural integrity and good rheology of the final hydrogels and controlling the rate of drug release at the site of inflammation, in vitro antibacterial activity caused by amikacin release, reached 90% for *Staphylococcus aureus* and 98% for *Pseudomonas aeruginosa* [125].

Innovative hydrogel is laden with antibiotics (DAC^®^, “Defensive Antibacterial Coating”), such as, among others, ceftazidime was tested in patients with periprosthetic infection of the hip joint. It was shown that the use of antibacterial hydrogel coatings loaded with the antibiotic might shorten the hospitalization time of patients and bring new solutions in the treatment of infections associated with implants in the field of orthopedics [126,127]. Defensive Antibacterial Coating, DAC is also a promising way of fighting post-operative site infection after internal osteosynthesis for closed fractures [128].

A hydrogel with high antibacterial activity against four various bacteria was obtained by Hu et al. by crosslinking oxidized dextran with simultaneous loading with two antibiotics: tobramycin and ornidazole. The injectable hydrogel exhibited thixotropic and self-healing properties and was acid-responsive due to the contribution of acid-labile Schiff base linkage during gel formation. The tobramycin-loaded hydrogel gel showed high antibacterial activity against aerobic pathogens (*Staphylococcus aureus* and *Pseudomonas aeruginosa*). However, it was less effective against anaerobic ones (*Clostridium sporogenes* and *Bacaeroides fragilis*). Interestingly, the ornidazole-loaded hydrogel showed opposite outcomes [129].

As can be seen from the concisely discussed bibliography presented above (including especially the recent literature reports), natural hydrogels are commonly tested as carriers of a wide range of antibiotics. Strategies for introducing antibiotics into hydrogel formulations include, among others, their physical loading, pre-adsorption on carriers (e.g., nanoparticles), or encapsulation in micelles, which are later dispersed in the hydrogel. What can be noticed is that the majority of research focuses only on in vitro demonstration of antibacterial activity; in vivo studies (including, for example, animal models) are rare. 

#### 2.2.2. Hydrogels Loaded with Biological Extracts or Natural Compounds

**Figure 5:**
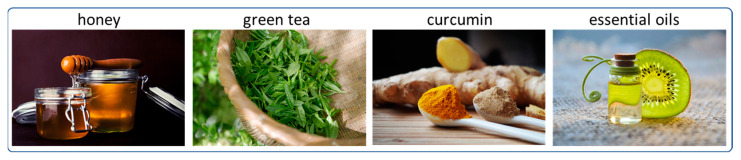
The most popular biological extracts used in antimicrobial hydrogels.

Hydrogels, as soft and biocompatible carriers, can also be used to deliver a whole range of plant-derived ingredients and their mixtures. These biological mixtures/extracts are characterized, on the one hand, by usually low general toxicity, and on the other, by unusual and often unexplored biological activity [130]. Often biological extracts have excellent biocidal properties. Therefore their incorporation in the hydrogel matrix is a very good opportunity to control and tune these properties. However, their therapeutic efficiency is often limited by various factors, including the lack of targeting capacity and poor bioavailability [131]. Biological extracts may come from plants and animals; some of these extracts have a long history of well-documented applications, while others were discovered in recent years [130,132,133]. Herbal extracts loaded within a hydrogel matrix may affect their structural, biological, and functional properties [133]. A whole range of compounds of natural origin possess biological attractiveness and have antibacterial properties making them potentially useful in various pharmaceutical and biomedical preparations [134,135] as their use is associated with antibacterial activity. Anyway, they are considered safe and thus can compete with antibiotic preparations [136]. The most studied families of chemical compounds derived from biological extracts include inter alia alkaloids, flavonoids, terpenoids and tannins, polyphenols, and their most frequently chosen sources are shown in Figure 5.

Due to its antimicrobial activity, topical nutrition, debriding action, minimalization of inflammation, stimulation of angiogenesis, granulation, wound contraction, and epithelialization, honey has been incorporated into wound dressings, many of them available commercially. The antimicrobial activity of honey is related to its acidity, low water content, and presence of a wide range of antimicrobial components, including hydrogen peroxide, antibacterial peptide defensin-1, flavonoids, and phenolic acids [137].

Gelam honey was incorporated into an agar-based hydrogel formulation, which was then cross-linked and sterilized using electron radiation 25 kGy. Its antibacterial activity against *Staphylococcus aureus* was studied with the conclusion that the topical application of the obtained formulations might have a favorable influence on the various phases of burn wound healing [138].

Injectable self-healing chitosan-based hydrogel with inherent antibacterial activity were fabricated by Zhong et al.by the dynamic boronate ester cross-linkages. At the same time, in-situ encapsulation of epigallocatechin-3-gallate, a green tea derivative, was successfully achieved. The resulting hydrogels showed dual bioactivity, i.e., antibacterial (against *Escherichia coli* and *Staphylococcus aureus*) and antioxidant, as well as good biocompatibility. Additionally, the in vivo wound healing studies using the skin model revealed the good performance of the hydrogels, showing their potential as wound dressings [139].

Feng et al. obtained composite hydrogel based on PVA, chitosan, and tannic acid by cryogenic treatment and freeze-drying method (cf. Figure 4), which let them obtain highly porous hydrogel with favorable swelling due to higher absorption capacity. It was shown that the addition of tannic acid improved the antibacterial activity of hydrogel, which was tested against *Escherichia coli* and *Staphylococcus aureus* [140]. In another study, the pH-controlled release of tannic acid and its antibacterial activity against *Escherichia coli* was investigated by Ninan et al. by its incorporation into agarose scaffolds cross-linked with zinc ions [141]. Interestingly, the obtained biomaterials displayed comparable antibacterial activity to one of the classic antibiotics—gentamicin.

Essential oils are distilled or extracted from various plants and are used in the food, medicine, and cosmetics industries due to their well-known antibacterial properties. The most abundant and, at the same time, the key components of essential oils are terpenes, which provide antibacterial properties as a result of well know bacteria-killing mechanisms [67]. Immobilization of essential oils increases their stability, bioactivity, and antibacterial potential but also reduces the volatility of essential oils.

Hydrogels based on carboxymethyl chitosan were synthesized and loaded with selected essential oils: eucalyptus essential oil, ginger essential oil, and cumin essential oil to prepare antibacterial materials. Among the developed hydrogels, the eucalyptus-loaded hydrogel exhibited optimal antibacterial activities of 46% against *Staphylococcus aureus* and 63% against *Escherichia coli*, along with high cell viability (>92%) and accelerated wound healing in mouse burn models by promoting the recovery of dermis and epidermis [142,143].

Mahmood et al. synthesized gellan gum hydrogels loaded with lavender oil and ofloxacin for wound healing application [144]. In vitro drug release studies showed sustained drug release of lavender oil (71%) and ofloxacin (85%) over the period of 48 h, while the in vitro antimicrobial analysis confirmed good antibacterial activity against Gram-negative (*Escherichia coli*) and Gram-positive (*Staphylococcus aureus*) bacteria, suggesting that these hydrogels can be potentially used as effective scaffolds for the treatment of infected wounds. Moreover, in vivo wound healing experiments using a full-thickness wound model on rats showed almost complete (98%) wound closure in loaded hydrogels when compared to the blank hydrogels [144].

Ionically crosslinked hydrogels, based on chitosan and poly(vinyl alcohol) loaded with tea tree oil and silver ions, were synthesized by Low et al. to demonstrate its feasibility for delivering the above-mentioned antimicrobial agents to treat common wound-infecting pathogens (*Candida albicans*, *Staphylococcus aureus*, *Psudomonas aeruginosa*) [145]. The tea tree oil contains over a hundred different components, including terpenes and associated alcohols, which heavily contribute to its broad spectrum antibacterial, antifungal, antiviral, and anti-inflammatory activities [146]. Therefore, combining tea tree oil and silver ions into the hydrogel matrix improved antimicrobial activity by lowering the required effective concentrations [145].

Altaf et al. tested poly(vinyl alcohol)/starch hydrogels loaded with tea tree oil, clove oil, and Oregano oil for wound dressing applications. In vitro antibacterial analysis of the loaded hydrogels showed good antibacterial activity against *Escherichia coli* and multi-resistant *Staphylococcus aureus*. The antibacterial result was highest for hydrogel loaded with clove oil, but unfortunately, the origin of this superior antibacterial activity was not elucidated [147].

Recently, the thyme oil-enriched cellulose-based hydrogels were investigated by Lu et al. exhibiting remarkable antibacterial activity against *Escherichia coli* and *Staphylococcus aureus*, suggesting their effectiveness as wound dressing materials for the treatment of bacteria-infected wounds. In vitro release test showed thyme oil burst release for the first 24 h followed by a slow and sustained release [148].

Thymol enriched bacterial cellulose hydrogel was tested as antibacterial dressing material, showing its effectiveness against a range of bacteria, including *Pseudomonas aeruginosa*, *Escherichia coli*, *Klebsiella pneumoniae* and *Staphylococcus aureus*. The incorporation of thymol into cellulose matrix has proved remarkable in vivo wound healing efficiency in rats with third degree burn wounds [149]. Research on various materials with incorporated thymol for applications as antibacterial wound dressing has been reviewed recently [150].

Curcumin is a biologically active substance extracted from turmeric, and it has high antioxidant activity. It has an analgesic effect and anti-inflammatory and anti-cancer properties. It has been tested in vitro and in vivo on curcumin-loaded wound dressings. Outcomes demonstrated therapeutic effects [151,152,153]. The commonly known curcumin as an anti-inflammatory and antioxidant added to hydrogel materials improves the wound healing process in diabetics [154,155].

Fathollahipour et al. prepared thermally crosslinked PVA-based hydrogels containing honey or sucrose for the purpose of erythromycin delivery. It was observed that the addition of honey to the hydrogel significantly slows down the release of the drug. Antibacterial tests showed the inhibitory action of erythromycin-loaded PVA hydrogels against *Pseudomonas aeruginosa* and *Staphylococcus aureus* [156].

The possible use of hydrogels loaded with herbal medicines has already been exploited in diverse areas, from product development to disease treatment, including confirmation of their translative potential in clinical trials [133]. For example, a randomized, double-blind, placebo-controlled clinical trial was conducted in Mexico with ambulatory patients [157]. The extract of the *Mimosa tenuiflora*(a popular remedy used in Mexico for skin lesions treatment)was incorporated into a composite hydrogel for the treatment of venous leg ulceration disease. It was shown that the size of the ulcer in patients treated with the extract-loaded hydrogel had been reduced, while those treated with the non-loaded hydrogel exhibited no significant improvement.

Hydrogels based on carboxymethylcellulose with loaded grapefruit seed extract have shown amazing antimicrobial activity. The results of the research prove that nanocomposite films increase the biocidal activity against *Escherichia coli* and *Staphylococcus aureus* [158].

The addition of carrageenan (natural linear sulfated polysaccharides usually extracted from red algae) to various hydrogel matrixes, including agar, alginate, chitosan, and gelatin, has proven to have a positive outcome in the treatment of difficult-to-heal-wounds-and-non-healing diabetic ulcers area of skin [159,160,161]. Apart from applications of carrageenan in pharmaceutical and industrial applications (e.g., as an emulsifier, stabilizer, or thickening agent), its inflammatory and immunomodulatory properties make carrageenan a promising antibacterial agent.

Chrysin is a bioflavonoid naturally found in martyr flowers, passionflowers, some kind of geranium, silver linden, bee propolis, or honey [162]. Nanofibers of chrysin included in the hydrogels show anti-inflammatory, anticancer, and antioxidant properties [163,164]. Another flavonoid, hesperidin (present in vegetables and fruits), was successfully loaded into alginate/chitosan or dendritic-based– the resulting biomaterials showed angiogenic, anti-inflammatory, and antibacterial properties [165,166].

As seen from the presented survey on natural extracts/compounds-loaded hydrogel formulation, integrating herbal medicines with hydrogel scaffolds may become a promising and nature-based approach in antimicrobial treatment. Anyway, many herbal medicines and synthetic drugs (but not all) may be loaded together to provide a synergistic boost. Nevertheless, delivering herbal medicines using hydrogel vehicles may be more complicated than expected because—due to the complex matrix—more optimization is required before success can be achieved [133].

Histologic evaluation, however, has shown that the therapeutic effect of the extract-loaded hydrogel is not significantly different from that of the blank hydrogel. This reveals that the successful incorporation of herbal medicines into hydrogels for clinical use may necessitate careful optimization.

#### 2.2.3. Hydrogels Loaded with Inorganic Particles

Inorganic antibacterial materials such as silver (Ag), gold (Au), and copper (Cu) have been used for ages. All these elements were added to enhance the antibacterial activity in a particular formulation. Zinc oxide (ZnO), titanium dioxide (TiO_2_), or nickel oxide (NiO) was also used for this purpose. The placement of such nanoparticles showed a low degree of toxicity.

In nature, silver occurs in the free state and in minerals, but most of the silver mined is mixed with copper, gold, zinc, and lead ores. In the early 18th century, silver nitrate was used for the treatment of infected ulcers or burn wounds [167]. Interestingly, high concentrations of silver performed to interact with skin cells, which are the necessary concentration to alter cellular respiration, are 25 times greater than that needed to inhibit the growth *Pseudomonas aeruginosa* [168].

**Figure 6:**
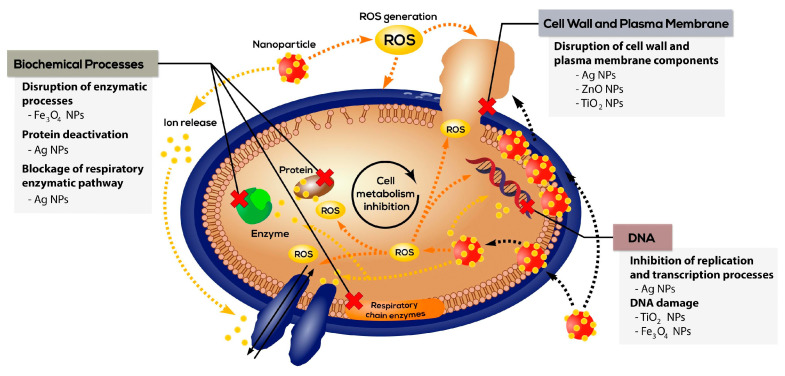
Various targets in the bacteria are available for nanoparticles, particularly silver nanoparticles. Reprinted with permission from [68]. Copyright 2022, Elsevier.

There are many medications available on the pharmaceutical market for treating wounds, especially silver-coated dressings or disinfectants [169]. Silver nanoparticles (Ag NPs)are a substitutive active ingredient for traditional methods of antibiotic therapy. They have a large surface area to volume ratio and thus are active at negligible concentrations showing a high antibacterial potential [170]. With the rise of resistance to commonly used antibiotics and disinfectants, scientists are responding to the threat by developing new antimicrobial materials to prevent or control infections caused by these pathogens. Inorganic antimicrobial agents are based on the antimicrobial nature of metals or metal oxides such as silver, gold, zinc, and copper, which are incorporated into hydrogels by physical adsorption and mixing. It is assumed that the antibacterial properties may result from the formation on the surface of metals or their oxide’s highly reactive oxygen forms (ROS). When ROS are formed, bacteria are eliminated. This is related to the formation of oxidative stress, oxidative lesions, and membrane lipid peroxidation [171,172,173]. Another hypothesis says that the irregular edges of the nanoparticles damage the structure of the bacteria, as a result of which it degrades [174]. Nanoparticles can also affect the inhibition of ATPase activity (reducing the amount of ATP), which results in damage to bacteria as a result of the body’s reactions. Their occurrence is dictated by the combination of ionized forms of metal elements with proteins present in the body. The main problem turns out to be the low efficiency of using antibacterial metals and metal oxides in aqueous solutions. An attempt has been made to combine antimicrobial agents with natural hydrogel materials (collagen, gelatin, fibroin, and keratin) that are compact and stay in place at the wound site [175,176]. Thanks to their porous structure, they are highly permeable and can store and release medicinal substances at the desired rate. In addition, hydrogels are highly biocompatible and can be applied directly to the wound [4]. The main metal ions that have antibacterial properties are silver, gold, zinc, copper, mercury, and cadmium ions. Recently, metal oxides in the form of nanoparticles, such as titanium dioxide (TiO_2_), zinc oxide (ZnO), nickel oxide (NiO), and iron oxide (Fe_3_O_4_), have also gained a lot of interest because metal oxides in inorganic nanoparticles can interact with the surface of the material. At the forefront of antibacterial agents are silver ions, which are 1000 times stronger than zinc and copper ions [177,178]. The main bacterial targets of silver nanoparticles (along with gold and magnetite ones) that have been used in the treatment of skin infections are schematically shown in Figure 6 [68].

Silver in this field is used in the form of nanoparticles, silver salts, and metallic silver. The antibacterial effect of AgNPs has been tested and confirmed by many scientists [179,180,181,182,183,184,185,186,187,188,189,190,191,192]. As a result of studies, it turned out that silver nanoparticles effectively destroyed such types of bacteria as *Staphylococcus aureus, Pseudomonas aeruginosa, Escherichia coli, Bacillus subtilis, Vibrio cholera, Salmonella typhi, Enterococcus faecalis* and others [193,194,195,196,197]. Shivastava et al. tested the multi-drug-resistant bacteria of the *S. typhi* group by growing them on LB agar plates that had been enriched with silver nanoparticles [198]. The presence of AgNPs resulted in the inhibition of bacterial growth by 60% at a concentration of 5 μg/mL and by 90% at a concentration of 10 μg/mL. Complete inhibition of bacterial growth was observed at a concentration of 25 μg/mL. Anisha et al. developed an antimicrobial sponge composed of chitosan, hyaluronic acid, and AgNPs to treat diabetic foot disease [199]. The obtained results indicate that such a hydrogel dressing can be used in the treatment of diabetic foot disease infected with antibiotic-resistant bacteria. Similar studies were conducted by Ruffo et al., synthesizing a biocompatible hydrogel called HyDrO-DiAb for the treatment of diabetic foot ulcers [200]. Other studies have shown that silver nanoparticles are a good alternative to silver sulfadiazine in the treatment of burns and do not cause toxic effects [201,202,203]. The effect of hydrogel dressings containing silver nanoparticles in their structure on the speed of healing burn wounds was studied by Boonkaew et al. [204], who checked the effectiveness of two dressings—Acticoat and PolyMemSilverW in comparison with self-made hydrogel dressings containing AgNPs. Their results showed high effectiveness of the hydrogel dressing (comparable to Acticoat) against most pathogens studied (*Pseudomonas aeruginosa, Acinetobacterbaumannii, Candida albicans, Staphylococcus aureus,* and *E. faecalis*), in contrast to the tested PolyMemSilverW, which demonstrated lower antibacterial activity.

In the research work of Mi et al., the effect of a two-layer dressing made of chitosan and enriched with AgNPs was investigated [205]. In vitro and in vivo tests confirmed the antibacterial effect of the dressing. Moreover, the hydrogel dressing had a high air permeability coefficient, which additionally accelerated the wound healing process. Ag NPs, in combination with guar gum-based hydrogels, exhibited strong antimicrobial activity and cytocompatibility [206,207]. Unfortunately, often the problem with AgNPs is that large amounts are released too quickly, which makes silver, despite its antibacterial properties, dangerous to health. To avoid side effects, Ag NPs were only used topically, so the antibacterial effect was negligible [208]. One study developed a hydrogel made of very short peptides that have the ability to combine into larger structures and form hydrogels [197]. Silver nanoparticles were synthesized in situ using UV radiation. Such a process made it possible to control the amount of AgNPs released—prolonged release for 14 days, and effective inhibition of bacterial growth was observed. The problem of too-high toxicity can be solved by the use of modified silver nanoparticles and the synthesis of silver nanoparticles as a result of in-situ reduction or supramolecular complexation. Rui Liu et al. designed a complex hydrogel consisting of gelatin and cellulose modified with –COOH groups; after the synthesis, silver nanoparticles were incorporated within the hydrogel [209]. Higher effectiveness of antibacterial ions was observed as a result of a prolonged process of silver-controlled release [210]. The goal of Haidari et al. was to introduce small silver nanoparticles into the interior of a temperature-sensitive hydrogel based on block copolymer F127. A high level of dispersion of ultra-small silver particles in the cross-linked structure of the hydrogel was obtained by depositing Ag NPs on two-dimensional structures (e.g., on graphene). The high dispersion and lower reactivity of the nanoparticles resulted in a much higher level of interaction with bacterial membranes, leading to damage to the biofilm produced by the bacteria and their death [211].

Jiang et al. tested the highly compatible polysaccharide glucomannan konjac in combination with chitosan and silver nanoparticles for use as a wound dressing [212]. Studies have proven the ability of this hydrogel structure to drain the wound from the fluid secreted by it, which visibly reduces inflammation. In addition, the hydrogel material itself reduced the toxicity of the silver nanoparticles themselves through their controlled release. The smart hydrogel proposed by Haidari et al. also had the ability to control the release of an antibacterial substance [213]. The AgNPs present in this structure were able to respond to pH changes—when the pH changed from acidic to basic, the system caused the release of the antibacterial substance (AgNPs) contained in the hydrogel. Studies have shown the effective elimination of gram-positive and gram-negative bacteria.

Gold nanoparticles, although they are less frequently used as antibacterial agents, still have a number of antimicrobial properties. Au NPs have the ability to connect to the bacterial cell membrane and get inside the bacteria, leading to its death. Brown et al. conducted a study that showed that gold nanoparticles alone do not have antibacterial properties, but in combination with ampicillin, they have the ability to eliminate bacteria that are resistant to drugs [214]. For example, methicillin-resistant bacteria of the *group S. aureus, P. aeruginosa, Enterobacter aerogenes,* and *E. coli*. Daniel-da-Silva et al. [215] developed a hydrogel made of gelatin charged with gold particles and then cross-linked with genipin. The formed structure, under the influence of external stimuli (in this case, temperature), encapsulated gold nanoparticles. In contrast to Ag NPs, gold nanoparticles turn out to be more beneficial for bone regeneration because they are not toxic to osteoblastic cells. This was proved in Ribeiro’s study by comparing the effect of the silk fibroin/nanohydroxyapatite hydrogel modified with Au NPs and Ag NPs [216]. It turns out that in the treatment of bones, antibacterial hydrogels can be used for all concentrations of Au NPs, while the safe concentration of Ag NPs is only 0.5 wt.%. Reddy et al. came up with the idea of combining Au and Ag nanoparticles in bimetallic (Ag, Au) hydrogel nanocomposites [217]. Their goal was to increase the antimicrobial activity of silver hydrogel nanocomposites. Varaprasad et al. even managed to create dual-metallic (Ag^0^-Au^0^) nanoparticles with mint leaf extract to obtain an antibacterial substance that was active against *Bacillus* and *E. coli* bacteria [218]. In another study, a dressing based on silver nanoparticles (Ag NP) was used to culture 3D fibroblast cells in vitro, where silver nanoparticles are released as aggregates and localized in the cytoplasm of fibroblasts [219]. Ag NPs have been proven to significantly reduce mitochondrial activity without damaging the cell.

Zinc and zinc oxide nanoparticles, similar to silver nanoparticles, are widely known for their antibacterial properties and are widely used in cosmetics for problematic skin care [220]. One of the mechanisms of action of zinc oxide is the formation of free radicals on the surface of ZnO NPs [221]. As a result of the reaction of free radicals with microorganisms, organic matter is oxidized to carbon dioxide, and bacteria are eliminated. A sprayable thermosensitive hydrogel containing a complex of zinc and metformin, which inhibits ROS production by activating autophagy, was used by Zhengwei et al. as a drug delivery system for the treatment of skin injuries [222]. On this basis, it can be concluded that the beneficial antibacterial effect of zinc oxide nanoparticles, the combination of which with biocompatible hydrogels, will facilitate direct application to the skin [223,224,225]. Majumder dealt with the preparation of a biomimetic hydrogel dressing on silk fabric in his research [226]. To give it biocidal properties, he decided to sonochemically cover it with ZnO nanoparticles. Studies suggest that the dressing has adequate mechanical properties and significant antibacterial properties. Moreover, phase-contrast microscopic studies showed that the adherence, growth, and proliferation of L929 fibroblast cells seeded on oxide nanoparticles functionalized on a hydrogel-grafted silk fibroin fabric dressing was much higher than that of pure silk fibroin [226]. In addition, tests were carried out on hydrogels that contained dual ionic cross-linked hydrogels in their structure [227,228]. On their basis, it was found that the complex hydrogel containing zinc and calcium ions facilitated the proliferation and migration of human fibroblasts and umbilical vein endothelial cells and thus supported the process of renewal of damaged epithelial cells and blood vessels.

The antibacterial properties of inorganic nanoparticles are of increasing interest. Among other nanoparticles that find wide antibacterial use in combination with hydrogels are TiO_2_, CeO_2_, CdSe, FeO, ZnS, and Cu [229,230,231,232,233,234,235,236,237,238,239,240]. In addition to silver, gold, and zinc nanoparticles, copper, for example, has biocidal properties. Giavaresi et al. conducted research on a soft hydrogel based on hyaluronic acid enriched with copper ions in the process of creating blood vessels [241]. Studies have shown an improvement in the healing of bones implanted during bone grafting as a result of the use of a new technique consisting in stimulating tissue vascularity with the use of biocompatible Hyal-50% material and copper ions. In a study by Villanueva et al., a starch hydrogel enriched with various concentrations of CuNPs and coated with a silica layer was shown to have antimicrobial activity against Gram-negative and Gram-positive bacterial species [242]. The antibacterial properties of said hydrogel have been proven for at least four cycles of use. Other studies have also confirmed the antibacterial effectiveness of hydrogels enriched with copper nanoparticles [242,243,244,245,246,247]. Iron oxides also have antibacterial properties. Sathiyaseelan et al. investigated the antimicrobial properties and wound healing rate using a chitosan/PVA nanocomposite sponge loaded with FeO nanoparticles. A significant increase in cell proliferation was demonstrated, and the tested CS/PVA-PD-FeO NPs sponge was found to be effective in the treatment of diabetic foot infections [248]. Moreover, many other scientists are studying the effect of iron oxide nanoparticles in hydrogel dressings with a controlled drug delivery system [249,250] and the effect on the stabilization of these hydrogels [251].

## 3. Conclusions and Future Perspectives

This review paper summarized the actual state of-the-art of various types of natural hydrogels used as antimicrobial materials. The growing number of publications on hydrogels with biocidal properties clearly shows that the advantages associated with their use in biomedicine are so important that they arouse the interest of many research groups in the world. Some of the scientific achievements have already been patented or successfully commercialized. Currently, commercial products are obtained mainly on the basis of hydrogels without antibacterial loadings; if there are any, they are usually silver nanoparticles. The latest innovations related to antimicrobial hydrogels’ fabrication address many urgent biomedical challenges, showing their remarkable potential to prevent or even combat various microbial infections. Various strategies, comprising functionalization with different groups or incorporation of antimicrobial agents (including antibiotics, natural products, and nanoparticles; all concisely discussed in this review article), were used to impart bactericidal properties to a plethora of various hydrogel-based formulations.

The vast majority of scientific work focuses on antibacterial hydrogels, while applications related to antiviral activity are very rare. This is probably due to the fact that research on viruses requires a stricter security regime, including access to higher biosafety levels laboratories (BSL-2 and BSL-3), while in the case of bacteria, lower biosafety levels are acceptable (BSL-1 and BSL-2). However, the ongoing COVID-19 pandemic will undoubtedly lead to more research into the antiviral properties of hydrogels.

Looking from a financial perspective, the biomaterial market is estimated to grow from 109 billion USD in 2020 to 216 billion USD by 2025 [252], which will definitely be a great incentive to turn basic research into ready-made solutions. One of the reported limitations hindering wider applications is the lack of enough strength to withstand the repetitive motion of the skin represents an important limitation [253]; therefore, the efforts on designing new hydrogels should also be focused on the simultaneous possibility of obtaining durable and self-healing hydrogels. Other important problems include [51]:

(i) the lack of clinical animal models, as most studied experimental animal models are healthy young animals—there is little discussion on some old animals or animals with diseases,
(ii) high susceptibility to damage during transport and storage, with possible drug leakage as well as deterioration of their structure and function.
(iii) Frequent lack of matching between hydrogel degradation rate, active ingredient release rate, and the wound regeneration rate.

Despite these temporarily invincible challenges, further development of simple, low-cost hydrogel-based antimicrobial biomaterials will undoubtedly be one of the key future directions of pharmaceutics and medicine.

## Data Availability

Not applicable.
